# Telitacicept versus belimumab in proliferative lupus nephritis

**DOI:** 10.3389/fimmu.2025.1715593

**Published:** 2026-01-19

**Authors:** Chang Wang, Man-Zhu Zhang, Xiao-Ying Yun, Ling-Xu Li, Jia-Wei Shan, Biao Liu, Bing Li

**Affiliations:** 1 Department of Nephrology, 2^nd^ Affiliated Hospital of Harbin Medical University, Harbin, China; 2 Department of Nephrology, Institute of Nephrology, 2^nd^ Affiliated Hospital of Hainan Medical University, Haikou, China; 3 Hainan Clinical Research Center for Urinary System Disease, Haikou, China; 4 National Health Commission of China, Key Laboratory of Tropical Disease Control, School of Tropical Medicine, Hainan Medical University, Haikou, China

**Keywords:** BAFF, belimumab, lupus nephritis, renal remission rate, telitacicept

## Abstract

**Background and objectives:**

Proliferative lupus nephritis (LN) is a severe pathological type of systemic lupus erythematosus with a high risk of progression to chronic kidney disease and end-stage renal disease. The prognosis of patients with proliferative LN has improved with advancements in treatment regimens. However, more effective molecular targeted therapies are still needed. This study aimed to evaluate the efficacy and safety of two novel biologics, telitacicept and belimumab, in the treatment of proliferative LN.

**Methods:**

Twenty-eight individuals diagnosed with proliferative LN (class III/IV ± V) were retrospectively enrolled at the Second Affiliated Hospital of Hainan Medical University between January 2021 and May 2025 and received either telitacicept (n=18) or belimumab (n=10) in conjunction with standard therapy for more than 24 weeks. The renal response rates were the evaluated outcome.

**Results:**

A total of 28 patients were enrolled, with 18 receiving telitacicept and 10 receiving belimumab. At 8 weeks, the telitacicept group presented a greater renal remission rate, with 8 patients (44.4%) achieving a complete renal response (CRR), 7 patients (38.9%) achieved partial renal response (PRR), and 3 patients (16.7%) showed no renal response (NRR). In contrast, only 6 patients (60%) achieved PRR in the Belimumab group. The renal remission rate at 8 weeks was significantly higher in the Telitacicept group compared to the Belimumab group (p=0.04). The telitacicept group also demonstrated greater improvements in the SLEDAI-2K score and complement C3 level. At 24 weeks, 72.2% of the telitacicept group achieved a CRR, whereas 60% of the belimumab group achieved a CRR, with no significant difference in renal response rates. Glucocorticoid and immunosuppressant use was successfully reduced in both groups. Additionally, both groups showed improvements in clinical parameters, but no significant difference was noted at 24 weeks.

**Conclusions:**

In this preliminary study, telitacicept appears to induce earlier renal and immunologic remission than belimumab, along with a potential reduction in the need for glucocorticoids and immunosuppressants. Further validation in larger studies is needed.

## Introduction

Systemic lupus erythematosus (SLE) is a chronic autoimmune disease that often leads to multiorgan inflammation and damage (1). Lupus nephritis (LN) is the most common complication, with a lifetime incidence ranging from 20% to 60% in patients with SLE. Approximately 10% to 30% of patients with LN progress to renal failure and require kidney replacement therapy (KRT) (2). Among them, proliferative LN (class III/IV ± V) is a severe pathological type, with the highest risk of requiring KRT if not promptly treated with effective therapeutic regimens, as it can lead to acute kidney injury (AKI) or chronic kidney disease (CKD) (3). Therefore, early and effective treatment is crucial for improving patient prognosis.

In recent years, with the progress in the study of the pathogenesis of SLE, many therapeutic targets have been explored, such as those that target B cells, T cells, and type I interferons (4). Among several B-cell-targeted strategies for treating LN, blocking B-cell activating factor of the TNF family (BAFF) or proliferation-inducing ligand (APRIL) has shown promising clinical potential. Belimumab is a monoclonal antibody that targets BAFF or B lymphocyte stimulator (BLyS) and is the first biologic agent approved for treatment of SLE (5). Telitacicept is a novel humanized recombinant TACI-Fc fusion protein that targets and neutralizes the activity of two cytokines, BAFF and APRIL. Telitacicept inhibits the development and survival of plasma cells and mature B cells, and it has been approved in China for the treatment of patients with active SLE (6). Optimization of treatment regimens to induce remission of proliferative LN and development of strategies aimed at reducing the steroid dosage remain challenges faced by clinicians. Although both telitacicept and belimumab are B-cell-activating factor/proliferation-inducing ligands, further research is needed to evaluate the clinical efficacy and potential risks of these two drugs as part of a multitarget treatment regimen for proliferative LN. Therefore, we conducted a single-center retrospective observational study to compare and analyze the efficacy and safety of telitacicept and belimumab in the treatment of proliferative LN.

## Methods

### Patients and study design

We conducted a retrospective observational analysis of 186 patients with LN diagnosed by renal biopsy at the Department of Nephrology, 2^nd^ Affiliated Hospital of Hainan Medical University, between January 2021 and May 2025. The inclusion criteria were as follows: 1. diagnosis of SLE on the basis of the 2019 European League Against Rheumatism/American College of Rheumatology (EULAR/ACR) classification criteria; 2. renal biopsy confirming proliferative LN (class III/IV ± V); 3. age ≥12 years old; and 4. at least 24 weeks of treatment with telitacicept or belimumab. The exclusion criteria included patients who had received other biologics (such as rituximab) within 24 weeks prior to enrollment and those with other immune diseases, severe infections, malignant tumors, or kidney transplants.

This study was approved by the Research Ethics Committee of the Second Affiliated Hospital of Hainan Medical University and was performed in accordance with the Declaration of Helsinki.

### Clinical and laboratory data assessment

Differences in laboratory parameters between the two patient groups were assessed at baseline, 8 weeks, and 24 weeks following the administration of telitacicept or belimumab. The evaluated parameters included plasma albumin, 24-hour urine protein, hematuria, estimated glomerular filtration rate (eGFR), and lupus activity indicators such as the SLE Disease Activity Index 2000 (SLEDAI-2K) score, anti-dsDNA antibody titer, and complement C3 and C4 levels. Serious adverse events that occurred during the observation period were also recorded.

### Outcomes

The primary outcome was the renal response at 24 weeks of treatment with telitacicept or belimumab, including complete renal response (CRR), partial renal response (PRR), and no renal response (NRR), as evaluated according to the 2019 “Chinese Guidelines for the Diagnosis and Treatment of Lupus Nephritis” (7). CRR was defined as normal 24-h urine protein (urine protein quantification <0.5 g/24 h or urine protein/creatinine ratio <500 mg/g), no active urinary sediment, a serum albumin concentration ≥35 g/L, and a normal or elevated SCr level of no more than 10% from baseline. PRR was defined as a reduction in urine protein by more than 50% from baseline, with urine protein quantification <3.0 g/24 h, serum albumin >30 g/L, and serum creatinine elevation not exceeding the level at baseline by 10%. NRR referred to cases in which neither complete remission nor partial remission was achieved. The secondary outcome included changes in the SLEDAI-2K score from baseline.

### Immunosuppressive treatment protocol

Belimumab was administered intravenously at a dose of 480 mg every two weeks for the first three doses, followed by once every four weeks, for a total treatment duration of 24 weeks. Telitacicept was administered by subcutaneous injection at a dosage of 160 mg once per week for a duration of 24 weeks. Patients could receive concomitant immunosuppressive therapy with glucocorticoids, antimalarials and other immunosuppressants as needed.

### Statistical analyses

Data analysis was performed using GraphPad Prism 9 and SPSS Statistics 26. Continuous variables that did not follow a normal distribution are presented as the median (Q1, Q3), where Q1 and Q3 represent the 25th and 75th percentiles, respectively, reflecting the distribution range of the data. Categorical variables are presented as percentages. Missing data were imputed using the median value. Group comparisons were performed using the Mann–Whitney U test. Rates in the two groups were compared using *Fisher’s exact* test. A *P* value < 0.05 was considered statistically significant.

## Results

### Baseline characteristics

**Figure 1:**
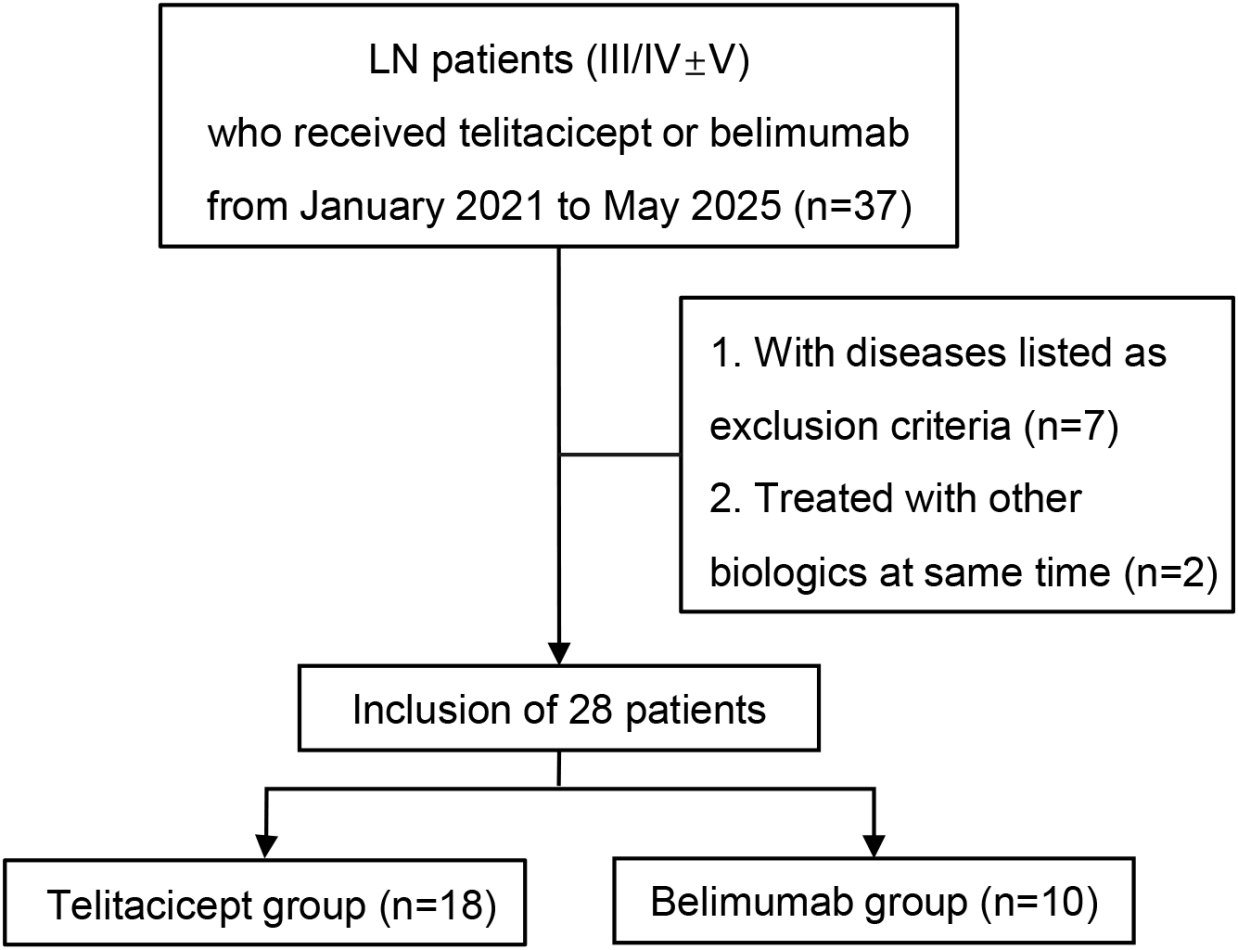
Research flow chart.

We reviewed 37 patients with LN (class III/IV ± V) treated with telitacicept or belimumab, 9 of whom met the exclusion criteria (**Figure 1**). A total of 28 patients met the inclusion criteria. Eighteen patients were included in the telitacicept group, and 10 patients were included in the belimumab group; relevant data on clinical information and treatment responses were analyzed (**Table 1**). The patients’ baseline characteristics, including age, sex, laboratory test results, and renal biopsy classifications, are detailed in **Table 1**. The proportion of males was relatively low in both groups, with 33.3% in the telitacicept group and 30% in the belimumab group. The median SLEDAI-2K score of patients in the telitacicept group was 16 (12, 18), whereas the median SLEDAI-2K score in the belimumab group was 15 (10, 16.5), indicating that SLE was in an active state at baseline. However, no significant difference between the two groups was detected (*p* = 0.49). Proliferative LN with membranous nephropathy was the primary renal biopsy finding in both groups (telitacicept group vs. belimumab group, 88.9% vs. 90%). All baseline characteristics, including body mass index, blood pressure, serum albumin, 24-hour urine protein, eGFR, serum creatinine, and complement C3 and C4 levels, were not significantly different between the two groups (*p*>0.05).

**Table 1:** Patient characteristics.

Characteristic	Telitacicept	Belimumab	*P* value
Number of Patients	18	10	–
Age	34 (22.8, 44.3)	26.5 (17.3, 37)	0.16
Male Sex, n (%)	6 (33.3%)	3 (30%)	
BMI	20.8 (19.1, 22.9)	19.9 (18.4, 22.1)	0.55
Blood Pressure (mmHg) Systolic BP Diastolic BP	129 (122, 138) 77.5 (72.5, 86.5)	129 (114.8, 134.3) 83 (75.3, 91.3)	0.59 0.31
ALB (g/dL)	2.4 (1.9, 2.8)	2.2 (1.8, 3.0)	0.49
Urinary Protein (g/24 h)	3.6 (3.5, 4.5)	3.9 (3.2, 5.3)	0.83
Hematuria (RBC/HPF)	35 (13, 105)	41.5 (30.8, 126.5)	0.35
Serum Creatinine (mg/dL)	1.0 (0.8, 1.2)	1.0 (0.6, 1.4)	0.83
eGFR (mL/min·1.73 m²)	76.4 (44.2, 100)	79.1 (51.9, 148.6)	0.49
WBC (10^9^/L)	5.4 (3.8, 7.1)	7.2 (4.3, 9.6)	0.25
Hb (g/L)	104 (83.8, 119.3)	105 (92.3, 120.5)	0.75
Plt (10^9^/L)	169.5 (127, 253)	287 (141, 367)	0.14
C3 (mg/dL)	40.5 (25.8, 61.8)	52.5 (48, 60.8)	0.27
C4 (mg/dL)	7.8 (6.6, 12.3)	7.4 (4.9, 12.3)	0.76
anti-dsDNA (IU/ml)	172.1 (65.9, 300)	172.5 (35.6, 298.5)	0.62
SLEDAI-2k score	16 (12, 18)	15 (10, 16.5)	0.49
Renal biopsy with V AI CI	16 (88.9%) 10.5 (7, 13) 2 (0, 3)	9 (90%) 10 (8.8, 11.3) 1.5 (1, 2.3)	0.94 0.94

ALB, albumin; BMI, body mass index; eGFR, estimated glomerular filtration rate according to the Cockcroft–Gault formula; Hb, hemoglobin; Plt, platelets; RBC, red blood cell; WBC, white blood cell.

Baseline: week 0 (at the start of telitacicept or belimumab treatment).

The data for both groups were obtained before infusion.

### Response to treatment

**Figure 2:**
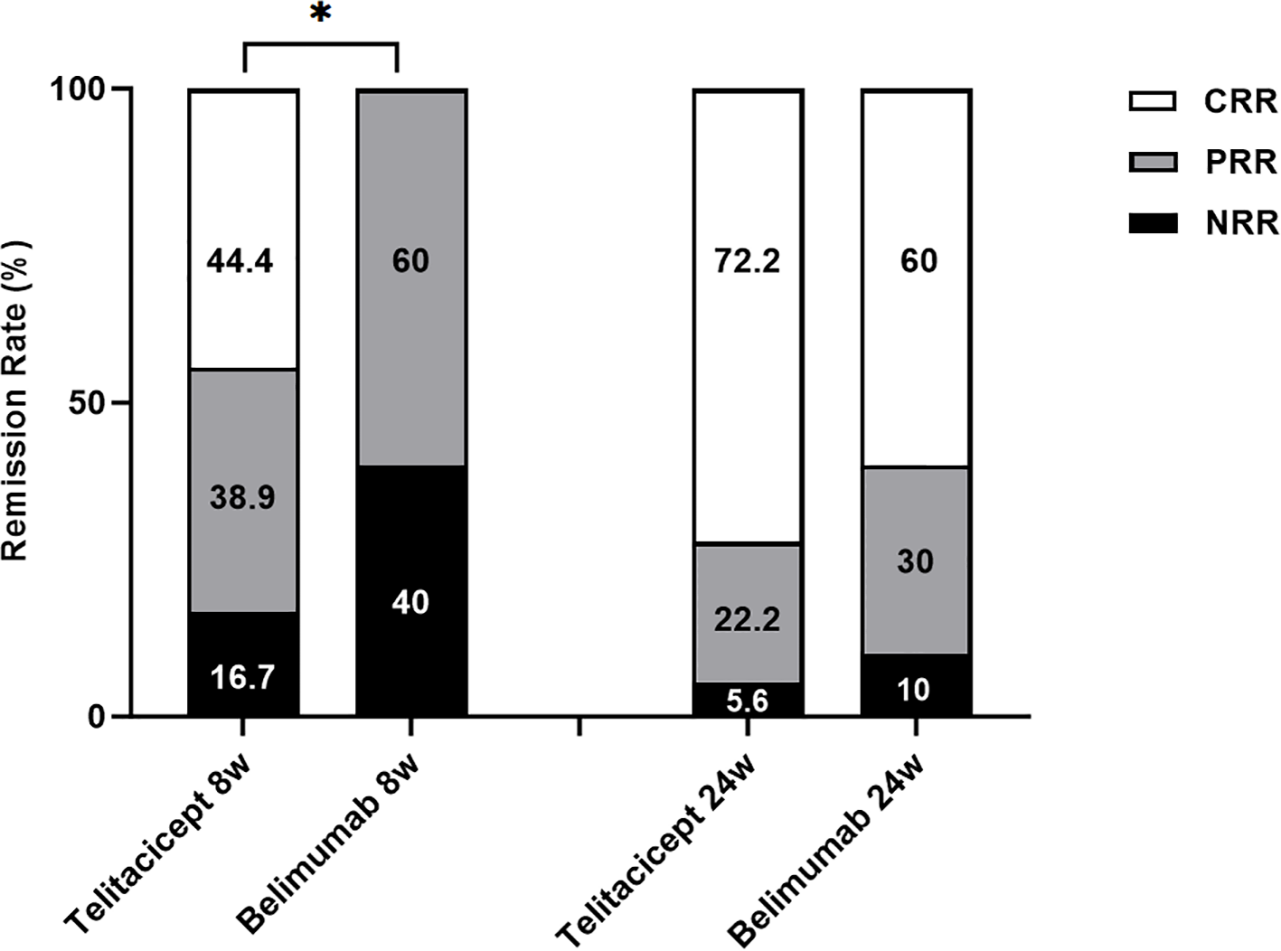
Rates of patients achieving renal remission in both groups during follow-up. *p<0.05.

During the follow-up period, both telitacicept and belimumab demonstrated favorable renal outcomes in the treatment of LN (**Figure 2**). At week 8, 8 patients (44.4%) in the telitacicept group achieved CRR, 7 patients (38.9%) exhibited PRR, and 3 patients (16.7%) had NRR. In contrast, 6 patients (60%) in the belimumab group achieved PRR, whereas the remaining 4 patients (40%) achieved NRR. Notably, at week 8, the telitacicept group demonstrated significantly better renal remission than the belimumab group did (*p* = 0.03). By week 24, 13 patients (72.2%) in the telitacicept group achieved CRR, 4 patients (22.2%) had PRR, and 1 patient had NRR. In the belimumab group, 6 patients (60%) achieved CRR, 3 patients (30%) had PRR, and 1 patient had NRR. By week 24, both groups exhibited improved renal response rates compared with baseline, and no statistically significant difference was observed between the groups (*p*>0.05).

### Reduction in corticosteroid and immunosuppressant use

**Figure 3:**
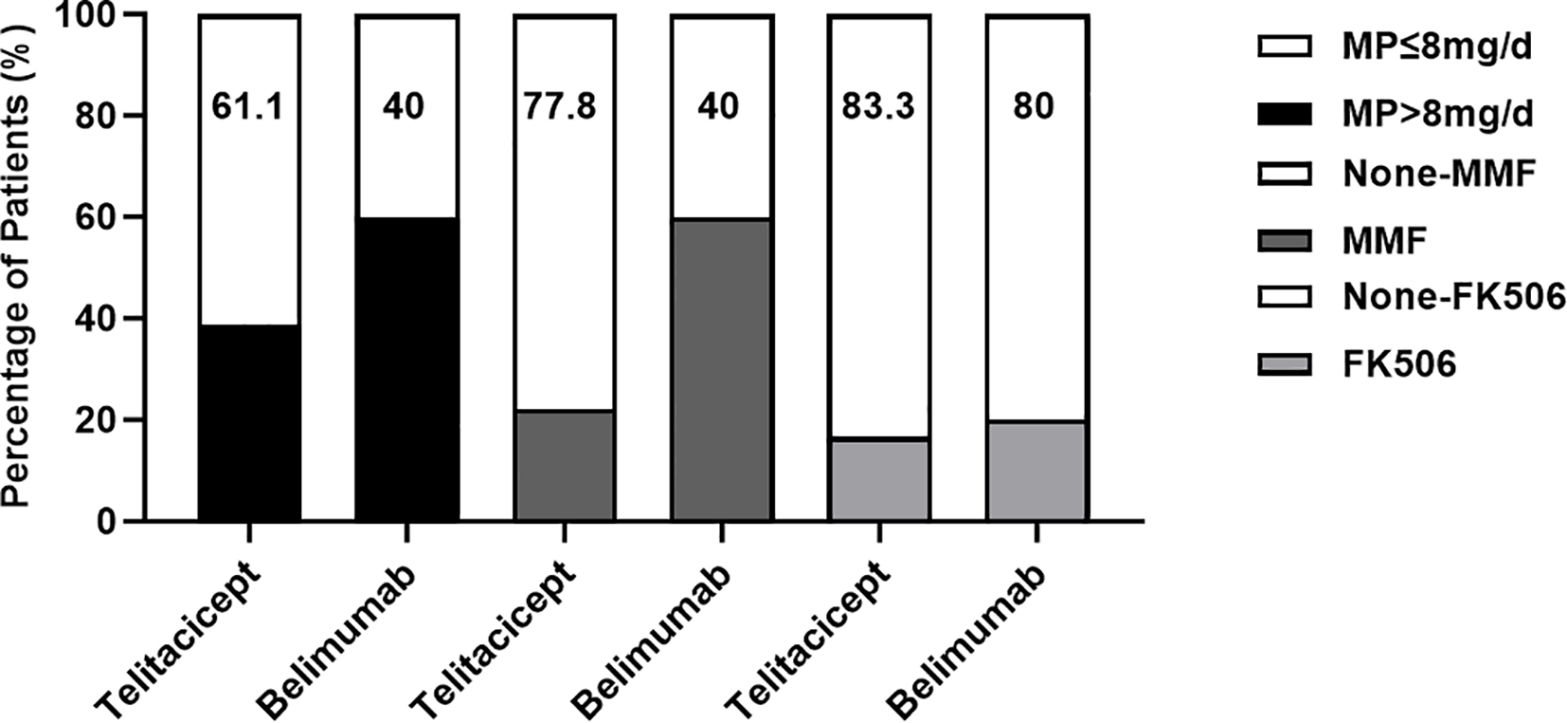
Percentages of patients using methylprednisolone and immunosuppressants in both groups at week 24. None-MMF, no use of MMF; None-FK506, no use of FK506.

At the final follow-up, both patient groups showed a reduction in the use of corticosteroids and immunosuppressants. In terms of corticosteroid use, 61.1% of patients in the telitacicept group used ≤8 mg/d methylprednisolone, whereas 38.9% used >8 mg/d. In contrast, 40% of patients in the belimumab group used ≤8 mg/d methylprednisolone, and 60% used >8 mg/d. Regarding immunosuppressant use, 77.8% of patients in the telitacicept group did not use mycophenolate mofetil (MMF), whereas 22.2% did. A greater proportion of patients in the belimumab group used MMF (60%) and 40% did not use it. Both groups demonstrated similarly low usage rates of FK506; it was not used in 83.3% of patients in the telitacicept group and 80% of patients in the belimumab group (**Figure 3**).

### Trends in clinical data changes

**Figure 4:**
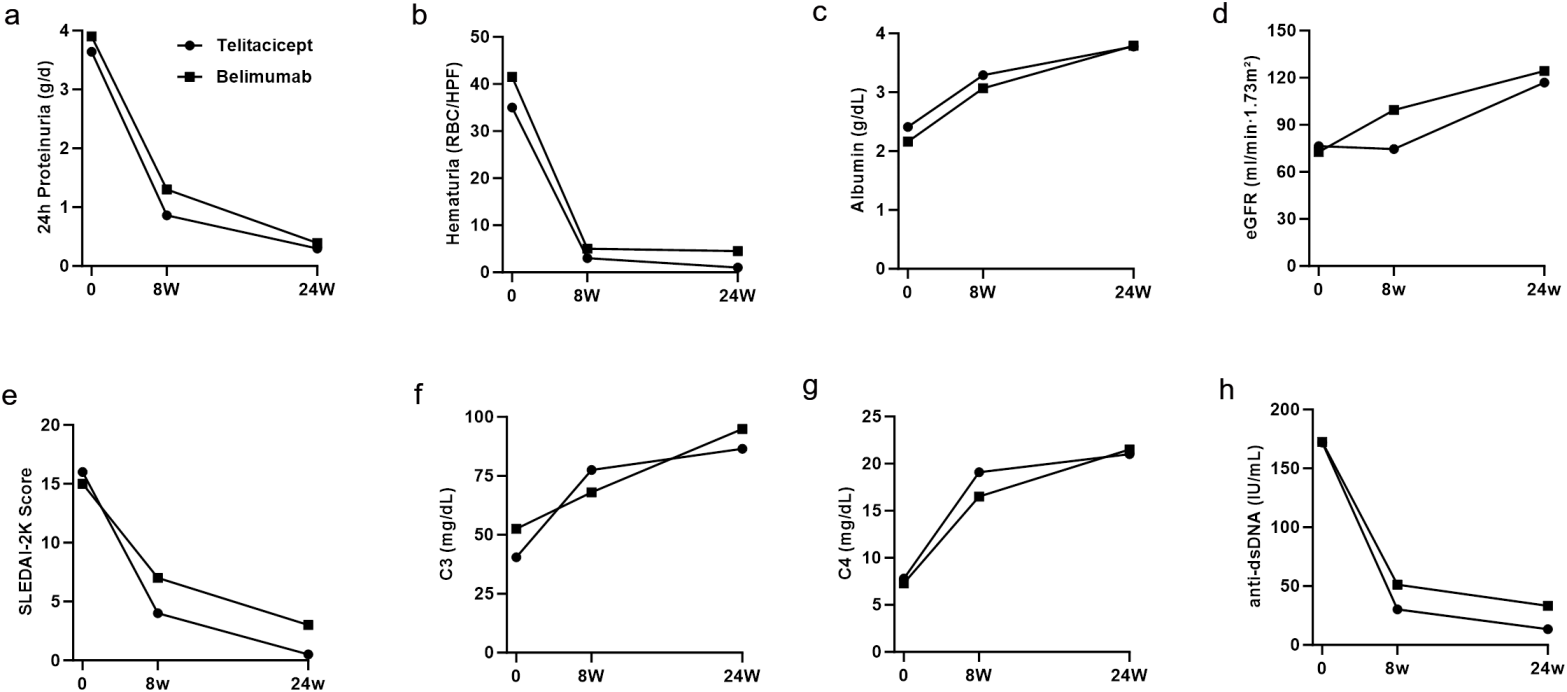
Dynamic changes in key parameters over 24 weeks: **(a)** 24-hour proteinuria, **(b)** hematuria, **(c)** serum albumin, **(d)** eGFR, **(e)** SLEDAI-2K score, **(f)** C3, **(g)** C4, and **(h)** anti-dsDNA levels in both groups.

The 24-hour urine protein levels (**Figure 4A**) and hematuria levels (**Figure 4B**) demonstrated notable decreasing trends, whereas the serum albumin level progressively increased (**Figure 4C**). Furthermore, the eGFR generally remained stable throughout the treatment period (**Figure 4D**). The median values of 24-hour urine protein, hematuria count, serum albumin, and eGFR at baseline, 8-week follow-up, and 24-week follow-up did not significantly differ between the groups (all *p*>0.05).

In terms of disease activity, both groups of patients improved after treatment. In the telitacicept group, the median SLEDAI-2K score decreased from 16 (12, 18) at baseline to 4 (2, 6.5) at week 8. In the belimumab group, the median SLEDAI-2K score decreased from 15 (10, 16.5) to 7 (5.5, 8.5) (**Figure 4E**). At week 8, the median SLEDAI-2K score in the telitacicept group was significantly lower than that in the belimumab group (*p* = 0.03). Furthermore, complement levels in both groups gradually returned to the normal range from baseline hypocomplementemia. At week 8, the median complement C3 level in the telitacicept group was 77.5 (71.8, 91) mg/dL, whereas in the belimumab group, it was 68 (58.5, 79) mg/dL (**Figure 4F**). At week 8, the median complement C3 level in the telitacicept group was significantly greater than that in the belimumab group (*p* = 0.04). Moreover, complement C4 (**Figure 4G**) and dsDNA (**Figure 3H**) levels also improved in both groups. At the final follow-up, the SLEDAI-2K score and complement C3, C4, and anti-dsDNA titers in both groups had returned to within the normal range, with no significant difference observed between the two groups (*p*>0.05).

### Severe adverse events

The severe adverse events recorded in both groups were primarily infections. In the telitacicept group, one patient developed purulent meningitis. In the belimumab group, one patient experienced severe lung infection, and another developed tuberculosis.

## Discussion

This retrospective comparative study adds to our existing knowledge on induction therapy for proliferative LN. In this study, 18 patients with Class III/IV (with or without Class V) LN received telitacicept treatment, and 10 patients received belimumab treatment. Both groups achieved favorable outcomes in terms of renal remission and a reduction in disease activity. To date, KDIGO, ACR, and EULAR all recommend a multitarget regimen as the preferred first-line induction therapy for patients with active, new-onset, or relapsing Class III/IV LN (8–10). Corticosteroids combined with immunosuppressants, including cyclophosphamide, calcineurin inhibitors, and MMF, remain the first-line treatment for proliferative LN. However, the long-term use of high-dose corticosteroids and immunosuppressants leads to more toxic side effects (11–14). Thus, current challenges in the treatment of proliferative LN include optimizing the cocktail immunosuppressive induction therapy regimen, reducing the steroid dosage, maintaining remission, preventing relapse, and preserving long-term renal health.

BAFF and APRIL are cytokines essential for the proliferation and survival of B cells during their development from the immature stage to the plasma stage. Two ligands, BAFF and APRIL, and three receptors, BAFF receptor (BAFF-R), B-cell maturation antigen (BCMA) and transmembrane activator and cyclophilin ligand interactor (TACI), form the backbone of the BAFF/APRIL system. Ligands exert their effects by binding to three receptors that play key roles in the pathogenesis of autoimmune disease (15, 16). Both BAFF and APRIL bind to BCMA, with APRIL exhibiting a relatively high affinity for BCMA (17), which plays a critical role in the survival of long-lived bone marrow plasma cells and plasmablasts (18). BAFF is overexpressed in SLE (19). Moreover, an immunohistochemical analysis of renal biopsy samples from patients with LN revealed elevated BAFF expression in the glomeruli of proliferative LN (20). Additionally, multiple studies have demonstrated that belimumab, a BAFF-targeting biologic agent, can improve renal outcomes (21–23). However, in clinical practice belimumab remains ineffective for some patients and has side effects (24, 25). Unlike belimumab, telitacicept simultaneously inhibits the binding of both BAFF and APRIL to their respective B-cell receptors. Notably, several clinical studies have demonstrated that telitacicept has high efficacy and good safety in patients with SLE (26–28).

Our study results indicate that, compared with belimumab, telitacicept has no significant difference in clinical efficacy or safety events in patients with proliferative LN while minimizing the use of glucocorticoids and immunosuppressants. Additionally, telitacicept has promising potential for the early induction of remission. Furthermore, belimumab is administered intravenously and requires hospitalization, whereas telitacicept is administered subcutaneously, offering greater convenience and suitability for outpatient treatment and improved patient adherence.

This study has limitations. It was a single-center retrospective study with a small sample size and a short follow-up period, which necessitate further validation through multicenter, randomized controlled trials. However, despite these limitations, this study may provide a new strategy for multitarget induction therapy for proliferative LN.

## Conclusion

This study provides preliminary evidence that telitacicept is a viable option for the treatment of proliferative LN. Compared with belimumab, telitacicept appears to induce earlier renal and immune remission while reducing the dosage of glucocorticoids and immunosuppressants, indicating its potential as a promising therapeutic option. Future prospective, multicenter, randomized controlled trials are urgently needed to validate these findings.

## Data Availability

The raw data supporting the conclusions of this article will be made available by the authors, without undue reservation.
